# Supplementary material for: A high-resolution mRNA expression time course of embryonic development in zebrafish
Source: eLife. 2017 Nov 16;6:e30860. doi: 10.7554/eLife.30860 (PMC5690287; doi:10.7554/eLife.30860)
Supplement: Supplementary file 6. [file elife-30860-supp6.zip › biolayout-clusters-files/Cluster158-genes.html]

Cluster158


# Cluster158: Genes

| Ensembl ID | Gene Name | Chr | Start | End | Biotype |
| --- | --- | --- | --- | --- | --- |
| ENSDARG00000014031 | abcc2 | 13 | 551337 | 581285 | protein\_coding |
| ENSDARG00000029381 | aldh3a2b | 21 | 38984761 | 39013538 | protein\_coding |
| ENSDARG00000045414 | elovl2 | 24 | 8720886 | 8747439 | protein\_coding |
| ENSDARG00000090228 | gsta.1 | 13 | 699322 | 722215 | protein\_coding |
| ENSDARG00000063481 | si:ch211-214j24.15 | 4 | 5295638 | 5308436 | protein\_coding |
| ENSDARG00000077872 | si:dkey-52d15.1 | 15 | 21231410 | 21241317 | unprocessed\_pseudogene |
| ENSDARG00000042188 | ttc38 | 18 | 10957393 | 10993420 | protein\_coding |
| ENSDARG00000055138 | zgc:110410 | 16 | 25684347 | 25693421 | protein\_coding |
| ENSDARG00000074684 | zmp:0000000758 | 14 | 47169775 | 47185962 | protein\_coding |
